# Complete Genome Sequence of *Mycobacterium bovis* Strain BCG-1 (Russia)

**DOI:** 10.1128/genomeA.00182-16

**Published:** 2016-03-31

**Authors:** Evgeniya A. Sotnikova, Egor A. Shitikov, Maja V. Malakhova, Elena S. Kostryukova, Elena N. Ilina, Alena V. Atrasheuskaya, Georgy M. Ignatyev, Nataliya V. Vinokurova, Vyacheslav Y. Gorbachyov

**Affiliations:** a Federal Research and Clinical Center of Physical-Chemical Medicine of Federal Medical Biological Agency, Moscow, Russian Federation; b Microgen, Federal State Unitary Scientific-Industrial Company for Immunobiological Medicines of the Ministry of Health of the Russian Federation, Moscow, Russian Federation

## Abstract

*Mycobacterium bovis* BCG (Bacille Calmette-Guérin) is a vaccine strain used for protection against tuberculosis. Here, we announce the complete genome sequence of *M. bovis* strain BCG-1 (Russia). Extensive use of this strain necessitates the study of its genome stability by comparative analysis.

## GENOME ANNOUNCEMENT

Bacille Calmette-Guérin (BCG), an attenuated strain of *Mycobacterium bovis*, was developed by Albert Calmette and Camille Guerin in 1921 as a vaccine against tuberculosis. As a result of distributing BCG throughout the world, daughter strains appeared, and one of the first strains was BCG Russia (1). It belongs to early strains and is one of the most widely used strains in the world (2). Though most of the described differences among BCG strains refer to the period before the worldwide use of lyophilized vaccine, Wada et al. (3) have shown that mutations in vaccine strains obtained by various vaccine manufacturers from a single cell source over time reach detectable levels. Because of that, whole-genome sequencing and comparison of all BCG Russia strains are extremely important. Here, we report the complete genome sequence of *M. bovis* BCG-1, isolated from a working seed lot used by NPO Microgen (Russia) for vaccine production.

Genomic DNA was isolated according to van Embden et al (4). A whole-genome shotgun library, prepared with a GS FLX Titanium rapid library preparation it (Roche 454 Life Science, USA), was sequenced using the GS FLX sequencer (Roche 454 Life Science, USA). The obtained 369,059 reads (245,806,603 nucleotides [nt]) were used for *de novo* assembly with GS De Novo assembler version 2.9, resulting in 81 large contigs. Then, a mate-pair library with insert sizes of 2 to 3 kb was sequenced with the Ion Torrent PGM sequencer (Applied Biosystems, USA); 546,890 reads were obtained (114,344,250 nt), which were used for scaffolding. Eleven scaffolds were generated by SSPASE software (5).

The complete genome sequence of *M. bovis* BCG-1 comprised one circular chromosome comprising 4,370,705 bp with an average GC content of 65.60%. Annotation was generated with the NCBI Prokaryotic Genome Annotation Pipeline. A total of 4,078 genes were predicted. Three rRNAs and 45 tRNAs were identified in the genome.

To confirm that the sequenced genome is a strain of BCG Russia, which was previously described, characteristics of the strain were analyzed. The following previously described features of BCG Russia were found: DU2 type I (6), 2 copies of IS*6110* (7), and RDRussia (8). A single-nucleotide insertion in the *recA* (9) gene was not found.

### Nucleotide sequence accession numbers.

The genome sequence of *M. bovis* strain BCG-1 (Russia) was deposited in GenBank under the accession number CP013741. The version described in this paper is the first version, CP013741.1.
