# Correction for Sotnikova et al., Complete Genome Sequence of *Mycobacterium bovis* Strain BCG-1 (Russia)

**DOI:** 10.1128/genomeA.00535-16

**Published:** 2016-06-02

**Authors:** Evgeniya A. Sotnikova, Egor A. Shitikov, Maja V. Malakhova, Vladislav V. Babenko, Elena S. Kostryukova, Elena N. Ilina, Alena V. Atrasheuskaya, Georgy M. Ignatyev, Nataliya V. Vinokurova, Vyacheslav Y. Gorbachyov

**Affiliations:** a Federal Research and Clinical Center of Physical-Chemical Medicine of Federal Medical Biological Agency, Moscow, Russian Federation; b Microgen, Federal State Unitary Scientific-Industrial Company for Immunobiological Medicines of the Ministry of Health of the Russian Federation, Moscow, Russian Federation

## AUTHOR CORRECTION

Volume 4, no. 2, e00182-16, 2016. Page 1: The byline and affiliation line should read as given above.

